# Effects of camptothecin derivatives and topoisomerase dual inhibitors on *Trypanosoma cruzi* growth and ultrastructure

**DOI:** 10.1186/1477-5751-13-11

**Published:** 2014-06-10

**Authors:** Otto Kischlat Lacombe, Aline Araujo Zuma, Camila Cristina da Silva, Wanderley de Souza, Maria Cristina M Motta

**Affiliations:** 1 Laboratório de Ultraestrutura Celular Hertha Meyer, Instituto de Biofísica Carlos Chagas Filho, Universidade Federal do Rio de Janeiro, 21491-590 Rio de Janeiro, RJ, Brazil; 2 Instituto Nacional de Ciência e Tecnologia em Biologia Estrutural e Bioimagens, Universidade Federal do Rio de Janeiro, Rio de Janeiro, RJ, Brazil; 3 Instituto Nacional de Metrologia, Qualidade e Tecnologia-Inmetro, 20261-232 Duque de Caxias, RJ, Brazil

**Keywords:** Cell proliferation, Kinetoplast, Nucleus, Topoisomerase inhibitors, Trypanosomatid protozoa, Ultrastructure

## Abstract

**Background:**

*Trypanosoma cruzi* is the etiological agent of Chagas’ disease that is an endemic disease in Latin America and affects about 8 million people. This parasite belongs to the Trypanosomatidae family which contains a single mitochondrion with an enlarged region, named kinetoplast that harbors the mitochondrial DNA (kDNA). The kinetoplast and the nucleus present a great variety of essential enzymes involved in DNA replication and topology, including DNA topoisomerases. Such enzymes are considered to be promising molecular targets for cancer treatment and for antiparasitic chemotherapy. In this work, the proliferation and ultrastructure of *T. cruzi* epimastigotes were evaluated after treatment with eukaryotic topoisomerase I inhibitors, such as topotecan and irinotecan, as well as with dual inhibitors (compounds that block eukaryotic topoisomerase I and topoisomerase II activities), such as baicalein, luteolin and evodiamine. Previous studies have shown that such inhibitors were able to block the growth of tumor cells, however most of them have never been tested on trypanosomatids.

**Results:**

Considering the effects of topoisomerase I inhibitors, our results showed that topotecan decreased cell proliferation and caused unpacking of nuclear heterochromatin, however none of these alterations were observed after treatment with irinotecan. The dual inhibitors baicalein and evodiamine decreased cell growth; however the nuclear and kinetoplast ultrastructures were not affected.

**Conclusions:**

Taken together, our data showed that camptothecin is more efficient than its derivatives in decreasing *T. cruzi* proliferation. Furthermore, we conclude that drugs pertaining to a certain class of topoisomerase inhibitors may present different efficiencies as chemotherapeutical agents.

## Introduction

The Trypanosomatidae family comprises protozoa of medical and veterinary importance. This group includes species that are the etiological agents of numerous human diseases, such as Chagas’ disease (caused by *Trypanosoma cruzi*), African sleeping sickness (caused by *Trypanosoma brucei*), and leishmaniasis (caused by *Leishmania* spp). Chagas’ disease was discovered in 1909 and nowadays affects about 8 million people in Latin America and new cases are being reported in non-endemic areas due to emigrations [1].

*T. cruzi* is a flagellated protozoan and like other trypanosomatids presents a single mitochondrion with an enlarged region, termed kinetoplast, which contains the mitochondrial DNA (kDNA). *T. cruzi* also has a single spherical nucleus presenting a condensed heterochromatin next to the nuclear envelope and around the nucleolus [2-6]. Since the nucleus and the kinetoplast are cellular compartments that contain DNA, their structural organization depends on enzymes such as topoisomerases, that play a key role during replication, transcription, recombination and repair [7-9].

DNA topoisomerases are classified into type I and type II. Type I attaches to DNA and breaks one strand of the double helix that can rotate around its own axis to revert supercoiling. On the other hand, type II binds to a DNA double strand and makes a gate allowing a second DNA double helix pass [10].

Several topoisomerase inhibitors have been developed based on different types of these enzymes that have been considered as potent targets in chemotherapeutic studies, especially with tumor cells. Topo I inhibitors, such as camptothecin, form a ternary complex, since they can trap the enzyme and DNA together [11-14]. Topo II inhibitors, such as mitoxantrone and etoposide, bind to the enzyme preventing the re-ligation of the DNA double strand. Furthermore, some inhibitors share characteristics of the two groups described above and target both topo I and topo II, thereby being called dual inhibitors [15].

Many topoisomerase inhibitors are natural products extracted from plants, such as camptothecin, isolated from *Camptotheca accuminata*, and several alkaloids, such as evodiamine, isolated from *Evodia rutaecarpa*[16]. Topotecan and irinotecan are camptothecin derivatives that have been used for ovarian and colorectal cancer treatments, respectively. These inhibitors target topo I and bind to DNA, forming a cleavable complex. The collision between this ternary complex and a replication fork generates DNA double-strand breaks, which may be related to the S-phase cytotoxicity, the G2/M cell cycle arrest and DNA damage that activates repair proteins [14].

Baicalein, luteolin and evodiamine are topoisomerase dual inhibitors. Baicalein is an alkaloid isolated from *Scutellaria baicalensis* used in the treatment of hypertension, atherosclerosis, dysentery and inflammatory diseases [16]. Luteolin is a flavonoid, a group of natural compounds with therapeutic properties that causes apoptosis in promastigote forms of *L. donovani*[17-19]. Evodiamine is an alkaloid extracted from *Evodia rutecarpa* used as an anticancer, anti-inflammatory and antiobesity agent [20]. This compound was initially classified as a topo I inhibitor, but then it was proposed that evodiamine could also bind to topoisomerase II [21].

In the present work, we evaluated the effects of the eukaryotic topoisomerase I inhibitors, topotecan and irinotecan, and the eukaryotic dual inhibitors baicalein, luteolin and evodiamine on the epimastigote forms of *T. cruzi*, considering its proliferation and ultrastructural organization.

## Materials and methods

### Protozoa culture

*T. cruzi* epimastigote forms were grown at 28°C for 24 h in liver infusion tryptose (LIT) medium [22] supplemented with 10% fetal calf serum.

### Drug treatment

Topotecan, irinotecan, baicalein, luteolin and evodiamine were purchased from Sigma Aldrich and diluted in dimethyl sulfoxide (DMSO) at 5 mM and 30 mM. The drug was added to the culture medium after 24 h of initial growth, which corresponds to the exponential phase. Drug concentrations were used as follows: 1, 5, 10, 50, 100, 200 and 300 μM. Every 24 h cells were collected and counted in a Neubauer chamber during the 96 h of cultivation. Paired t-tests were applied to the results using 95% confidence interval (GraphPad Prism version 5.00 for windows; GraphPad Software Inc., San Diego, CA).

Cell viability was performed using the MTS/PMS colorimetric method, which is based on dehydrogenase activity and the conversion of MTS into formazan, that indicates the number of metabolically active cells [23]. Parasites were incubated with MTS/PMS solution for 4 h and formaldehyde 0.4% was used as negative control. The percentage of viable protozoa was obtained through a spectrofluorimeter (Molecular Devices Microplate Reader (SpectraMax M2/M2^e^, Molecular Devices) using a 490 nm wavelength. MTS/PMS is a colorimetric assay, based on dehydrogenase activity and the conversion of MTS into formazan, that indicates the number of metabolically active cells.

### Transmission electron microscopy

Protozoa were fixed in 2.5% glutaraldehyde diluted in 0.1 M cacodylate buffer (pH 7.2) for 1 h at room temperature and were washed in the same buffer. Cells were post-fixed for 1 h in 0.1 M cacodylate buffer containing 1% OsO_4_ and 0.8% potassium ferricyanide. Protozoa were washed in the same buffer and were dehydrated in a graded series of acetone and embedded in Epon (Electron Microscopy Sciences, Hatfield, PA). Ultrathin sections were stained with uranyl acetate and lead citrate and were observed using a Zeiss 900 transmission electron microscope (Zeiss, Oberkochen, Germany).

## Results

**Figure 1:**
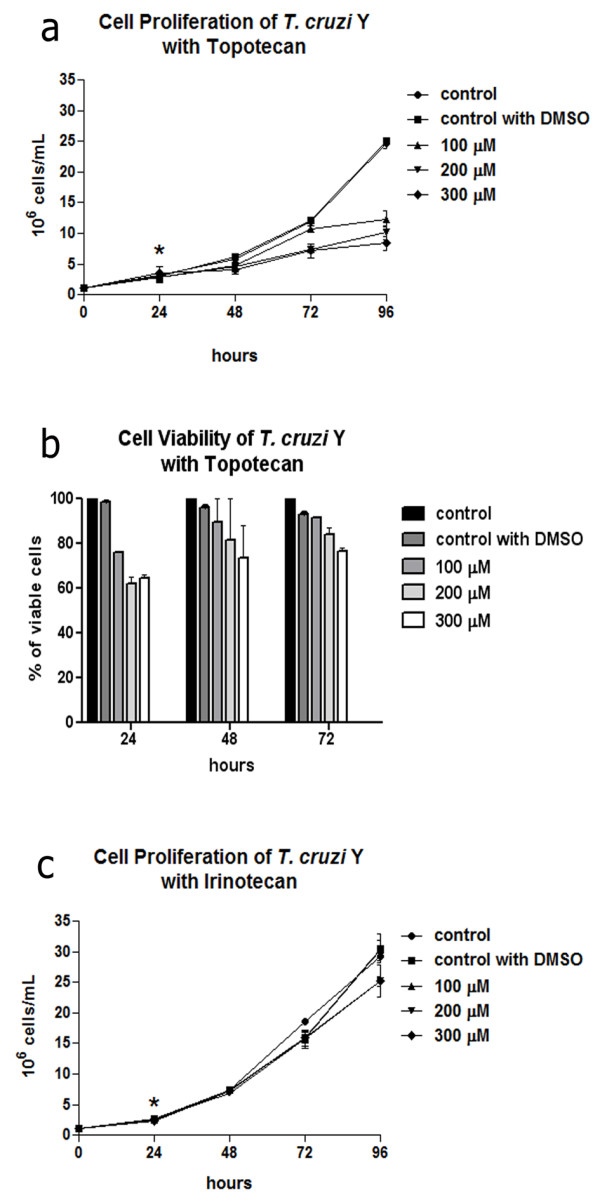
**The effects of topoisomerase I inhibitors on *T. cruzi *epimastigotes after 72 h of treatment. (a)** Topotecan affected cell proliferation especially after using higher drug concentrations. **(b)** Cell viability of *T. cruzi* treated with topotecan was reduced in a dose-dependent way after treatment for 48 h. **(c)** Irinotecan did not cause a significant growth inhibition even after treatment for 72 h. The asterisks indicate drug addition to the culture medium. Data are the average of three independent experiments. DMSO, dimethyl sulfoxide.

Regarding topoisomerase I inhibitors, cell proliferation was not significantly affected by topotecan after treatment with 50 μM for 72 h, while up to 300 μM reduced cell proliferation by approximately 3 fold in relation to the control cells was observed (Figure 1a), resulting in IC50 value of 110 μM. It is interesting to point out that induction of cell growth inhibition was noted after 48 h of treatment. Furthermore, this compound induced cell viability decay in a dose dependent manner, which corresponds to 20% after protozoa cultivation in medium containing 200 or 300 μM topotecan for 72 h (Figure 1b). On the other hand, irinotecan did not promote growth impairment with any of the concentrations tested (Figure 1c). Also cell viability was not affected (data not shown for Additional file 1).

**Figure 2:**
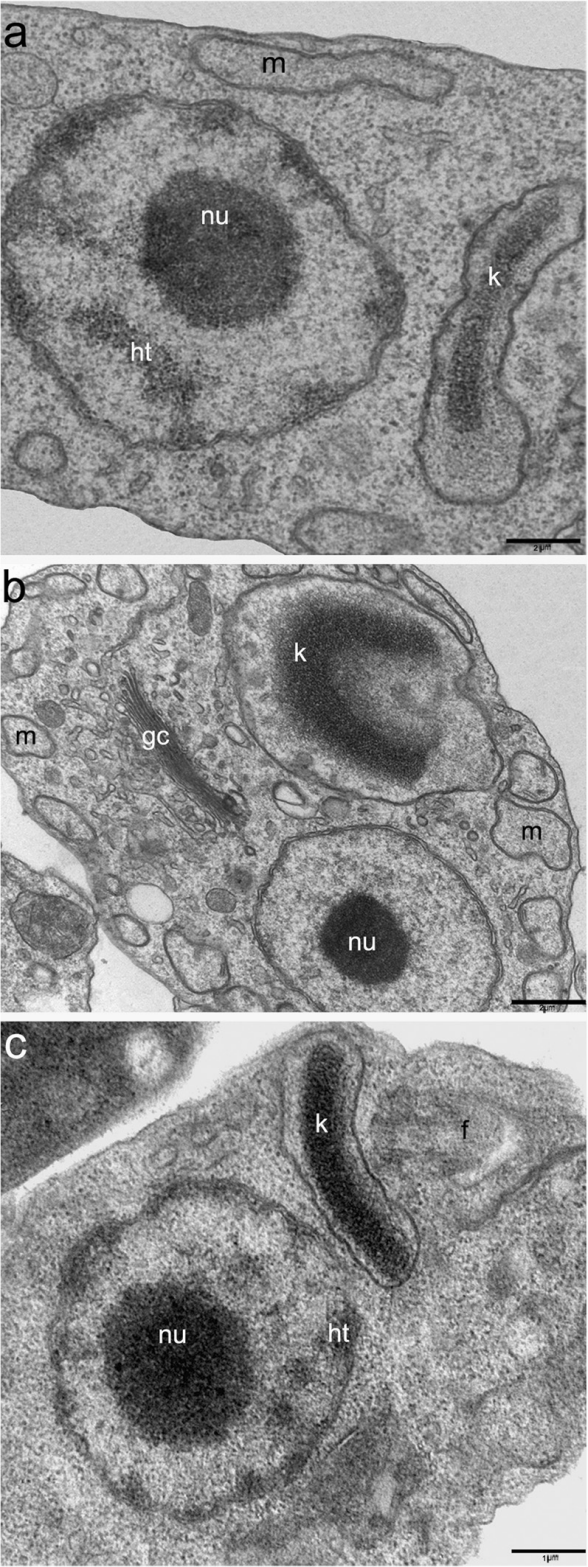
**The effects of topoisomerase I inhibitors on the ultrastructure of *T. cruzi *epimastigotes. (a)** Control parasite, showing the typical organization of the nucleus containing the condensed heterochromatin (ht) around the nucleolus (nu), the bar shape kinetoplast (k) and the mitochondrion (m). **(b)** When *T. cruzi* was treated with 300 μM topotecan for 72 h, the cells presented an unpacking of nuclear heterochromatin and mitochondrial swelling (m), especially in the kinetoplast (k) region. **(c)***T. cruzi* treated with 300 μM of irinotecan for 72 h, showing a slight unpacking of nuclear heterochromatin (ht). gc, Golgi complex; f, flagellum. (A) and (B) Bars = 2 μm. (C) Bars = 1 μm.

Transmission electron microscopy was used to study the ultrastructural modifications caused by these inhibitors. After treatment with lower concentrations, *T. cruzi* ultrastructure was similar to control cells (Figure 2a). However protozoa cultivation with 300 μM topotecan for 72 h led to an unpacking of the nuclear heterochromatin around the nucleolus and juxtaposed to the nuclear envelope. Mitochondrial swelling was also observed, especially in the kinetoplast region; however the kDNA arrangement was not affected (Figure 2b). Like the lack of effect in cell proliferation, irinotecan did not cause any significant changes in the cell ultrastructure. The main effect observed was a slight unpacking of heterochromatin (Figure 2c) and mitochondrial swelling, but with less intensity when compared to protozoa treated with topotecan.

**Figure 3:**
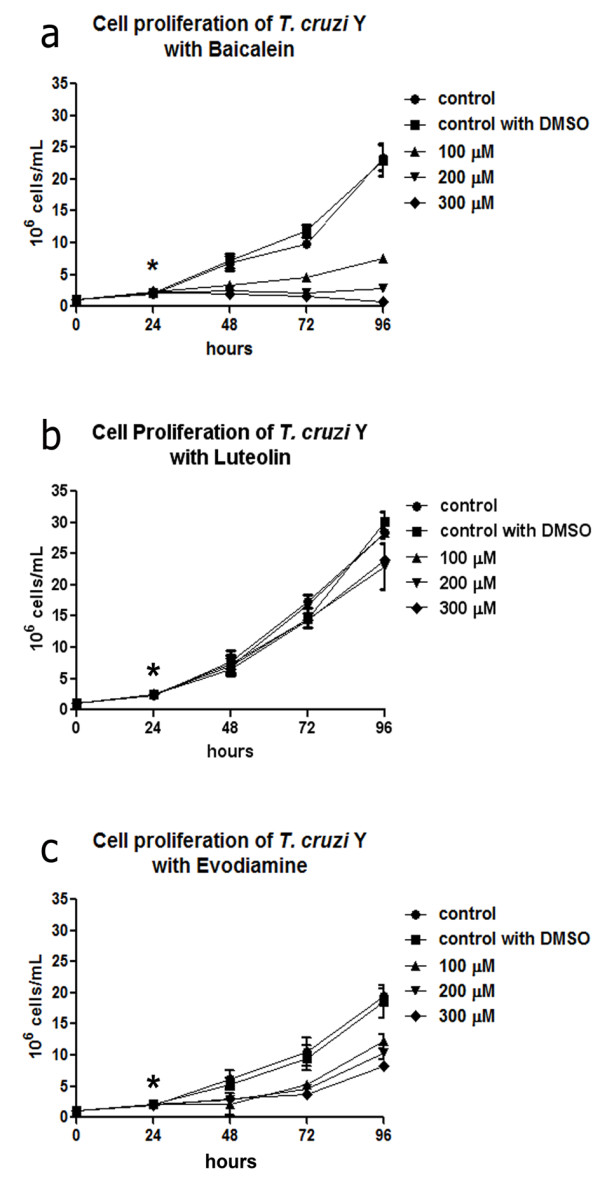
**The effects of dual inhibitors on *T. cruzi *epimastigotes after 72 h of treatment. (a)** Baicalein considerably reduced protozoa proliferation in a dose-dependent manner. **(b)** Luteolin did not promote growth impairment. **(c)** Evodiamine caused a reduction of parasite proliferation, but values were very similar with different drug concentrations. The asterisks indicate drug addition to the culture medium. Data are the average of three independent experiments. DMSO, dimethyl sulfoxide.

**Figure 4:**
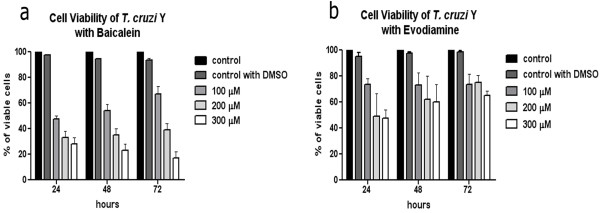
**The effects of baicalein and evodiamine on *T. cruzi *viability after 72 h of treatment. (a)** The number of viable cells was strongly reduced after treatment with baicalein. **(b)** Evodiamine caused a lower reduction of cell viability when compared to baicalein. Data are the average of three independent experiments. DMSO, dimethyl sulfoxide.

The dual inhibitor baicalein affected cell proliferation in a dose-dependent manner (Figure 3a), resulting in an IC_50_ of 62.83 μM after 72 h. Treatment with 300 μM baicalein for 24 h promoted a decrease of approximately 3 fold in parasite number and this difference increased to about 24 fold after 72 h (Figure 3a). Similar results were observed in cells treated with 200 μM baicalein, whereas the concentration of 100 μM reduced protozoa proliferation, but did not promote cell growth arrest. Luteolin was not able to inhibit protozoa growth significantly even after treatment with 300 μM for 72 h (Figure 3b). Evodiamine only promoted an expressive decay in cell proliferation after using concentrations equal or superior to 100 μM, when it was possible to observe a dose-dependent decrease in growth (Figure 3c), which is lower when compared to that caused by baicalein (Figure 3a). Regarding cell viability, after comparing the effect of these topoisomerase dual inhibitors, it is possible to conclude that baicalein was the most potent drug. This compound reduced the percentage of viable protozoa to approximately 30% after treatment with 200 μM for 24 h and this value was inferior to 20% after using 200 μM for 72 h (Figure 4a). The effect of evodiamine on cell viability was less intense than that observed for baicalein, but interestingly it was more pronounced in cells treated for 24 h (Figure 4b). Luteolin did not interfere in cell viability (data not shown for Additional file 2).

In terms of *T. cruzi* ultrastructure, the parasites treated with dual inhibitors did not present alterations when compared to the control cells. These compounds did not lead to modifications in the kDNA topology or in the heterochromatin organization, as was observed after treatment with topo I inhibitors (data not shown for Additional file 3).

Table 1 summarizes data obtained in this work showing the IC_50_ values and the main ultrastructural alterations caused by topoisomerase I and dual inhibitors to the *T. cruzi* epimastigotes.

**Table 1:** **Effects of topoisomerase I and dual inhibitors on *Trypanosoma cruzi *** **after 72 h of treatment**

**Drugs**	**Target**	**IC**_**50 **_**(μM)**	**Ultrastructural effects**
**Topotecan**	Eukaryotic Topo I Inhibitor	110	Unpacking of nuclear heterochromatin and mitochondrial swelling
**Irinotecan**	Eukaryotic Topo I Inhibitor	> 300	No modifications
**Baicalein**	Eukaryotic Dual Inhibitor	62,83	No modifications
**Luteolin**	Eukaryotic Dual Inhibitor	> 300	No modifications
**Evodiamine**	Eukaryotic Dual Inhibitor	90,73	No modifications

## Discussion

In the present work, the effects of different topoisomerase inhibitors were evaluated considering *T. cruzi* proliferation and ultrastructure. Irinotecan and topotecan are derivatives of camptothecin, thus they act by binding to DNA and to topoisomerase I by forming a ternary complex, referred to as a cleavable complex. These compounds interfere with the re-join of the double-strand break, leading to cell cycle blockade, activation of DNA repair and apoptosis [24].

Here, we observed that topotecan promoted a moderate effect on cell proliferation, whereas irinotecan did not affect protozoa growth. These results revealed that such inhibitors were not efficient in impairing *T. cruzi* growth when compared to camptothecin, the precursor compound, which presented IC_50_ values of 2.08 μM. The typical ultrastructural alterations, such as the unpacking of nuclear heterochromatin, promoted by topoisomerase I inhibitors were observed in cells treated with topotecan; however such modifications were only noticed after using high drug concentrations [25].

As described previously, topotecan and irinotecan were able to inhibit tumor cell proliferation, and were more effective and less toxic than camptothecin [26,27]. Such effects have also been reported on *T. brucei* and on *Leishmania infantum* promastigotes. In both these trypanosomatid species topotecan presented more efficacy than irinotecan, especially in *T. brucei*. The IC_50_ values correspond to 1.23 μM for topotecan and 21.5 μM for irinotecan on *T. brucei*, whereas values are equivalent to 10.86 μM for topotecan and superior to 200 μM for irinotecan on *L. infantum*[28,29]. A previous work also showed that camptothecin was cytotoxic to *T. brucei* and *L. donovani*, with IC_50_ values ranging from 1 to 3 μM [30].

Baicalein was the most effective compound against *T. cruzi* proliferation and viability considering all the inhibitors evaluated in this study. The treatment of *Leishmania* promastigotes with concentrations inferior to 15 μM of baicalein for 24 h was previously reported to reduce parasite growth up to 89% [31]. Furthermore, published data demonstrated that this drug inhibited tumor cell growth *in vitro* and *in vivo* and presented low toxicity [16,32].

Baicalein, evodiamine and luteolin are all classified as dual inhibitors of topoisomerase; but the latter compound did not promote any effect on *T. cruzi* cell proliferation. However, luteolin inhibits the growth of several cancer cell lines, blocking the cell cycle in the G1 phase [33]. In *Leishmania*, this inhibitor was also able to induce cell cycle arrest and apoptosis [18]. In this work, evodiamine promoted a slight inhibition of *T. cruzi* proliferation (IC_50_ 90 μM) when compared to baicalein (IC_50_ 62.83), however this compound has presented efficacy against different cancer cell lines [20]. Dual inhibitors target topoisomerases I and II, thus it was expected that such compounds could present high efficiency in blocking cell proliferation and also promoting ultrastructural changes in the nucleus and kinetoplast; however these effects were not observed in *T. cruzi* after treatment with these inhibitors.

## Conclusions

DNA topoisomerases represent an interesting target for anti-parasitic chemotherapy, since their inhibition interferes with the replicative process, which can lead to parasite death. In this work, we showed that compounds pertaining to the same topoisomerase inhibitor class had different effects on *T. cruzi* proliferation and ultrastructure. All inhibitors evaluated in this work are efficient for cancer therapy and sometimes blocked trypanosomatid growth, however their effects on *T. cruzi* proliferation and ultrastructure had never been investigated. Thus, we considered that they could be promissory agents in chemotherapeutic studies against *T. cruzi*, however these compounds presented considerably high IC_50_ values. The low effects observed in this parasite can be related to distinct factors such as the differences in human and protozoan topoisomerase domains, affinity for the target enzyme, cell membrane permeability and cell resistance, including mechanisms of drug efflux. Our results reinforce the idea that it is necessary to develop new compounds that may be successfully used in the therapy against neglected diseases.

## Competing interests

The authors declare that they have no competing interests.

## Authors’ contribution

OL carried out the proliferation inhibition, ultrastructural and cell viability assays. AZ participated in the proliferation inhibition, ultrastructural and cell viability experiments and drafted the manuscript. CS carried out the ultrastructural and viability assays. WS conceived of the study and participated in the design of the manuscript. MCMM conceived of the study, designed and drafted the manuscript. All authors read and approved the manuscript.

## Supplementary Material

Additional file 1 **Cell viability of *T. cruzi *** **Y with irinotecan.***T. cruzi* viability was not affected after treatment with irinotecan. The number of treated cells were similar to control parasites. Click here for file

Additional file 2 **Cell viability of *T. cruzi* Y with luteolin.***T. cruzi* viability was not affected after treatment with luteolin. Click here for file

Additional file 3 **The effects of dual inhibitors on the ultrastructure of *T. cruzi* epimastigotes.** (A) *T. cruzi* treated with 50 μM of baicalein for 72 h. (B) *T. cruzi* treated with 300 μM of luteolin for 72 h. (C) *T. cruzi* treated with 300 μM of evodiamine for 72 h. Note that the nucleus and the kinetoplast preserved their typical organization. Bars = 1 μm. K, kinetoplast; ht, heterochromatin; nu, nucleolus; m, mitochondrion; f, flagellum. Click here for file
