# Supplementary figures and images for: Effects of camptothecin derivatives and topoisomerase dual inhibitors on Trypanosoma cruzi growth and ultrastructure
Source: J Negat Results Biomed. 2014 Jun 10;13:11. doi: 10.1186/1477-5751-13-11 (PMC4066697; doi:10.1186/1477-5751-13-11)

# Cell Viability of *T. cruzi* Y with Irinotecan

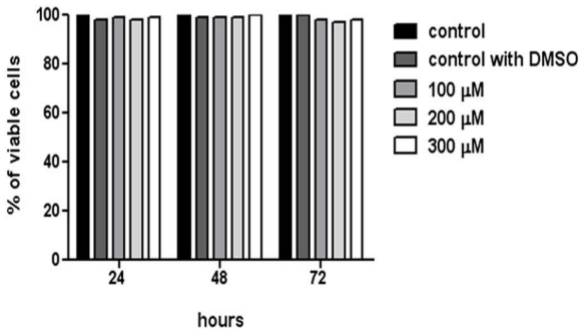

Supplement: Additional file 1 — Cell viability of T. cruzi Y with irinotecan.T. cruzi viability was not affected after treatment with irinotecan. The number of treated cells were similar to control parasites. [file 1477-5751-13-11-S1.pdf]

## Cell Viability of *T. cruzi* Y with Luteolin

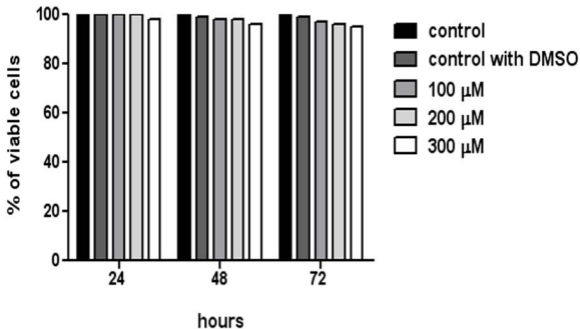

Supplement: Additional file 2 — Cell viability of T. cruzi Y with luteolin.T. cruzi viability was not affected after treatment with luteolin. [file 1477-5751-13-11-S2.pdf]

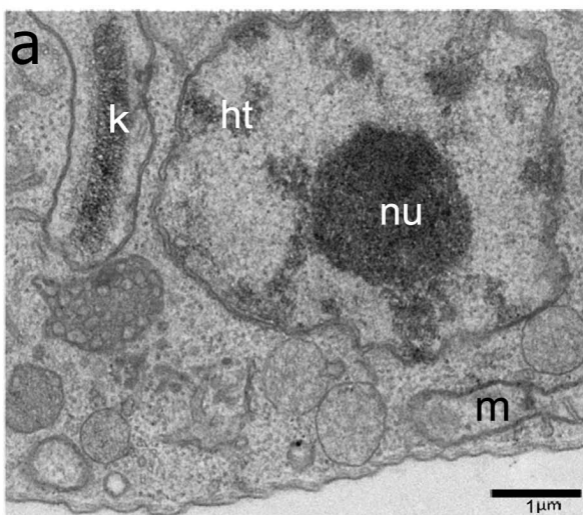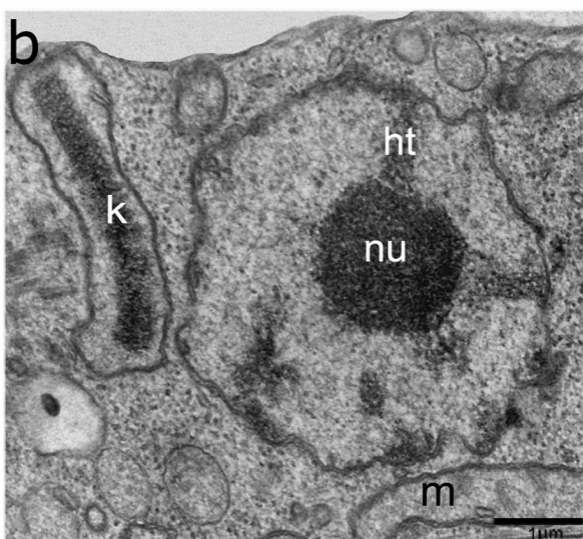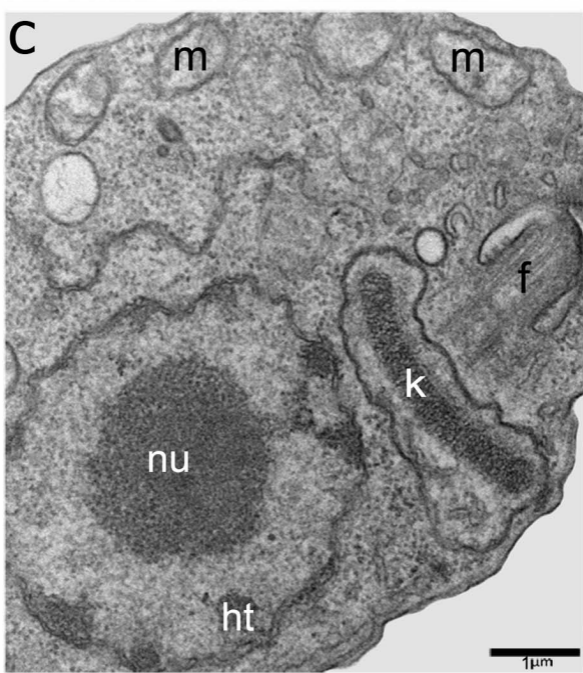

Supplement: Additional file 3 — The effects of dual inhibitors on the ultrastructure of T. cruzi epimastigotes. (A) T. cruzi treated with 50 μM of baicalein for 72 h. (B) T. cruzi treated with 300 μM of luteolin for 72 h. (C) T. cruzi treated with 300 μM of evodiamine for 72 h. Note that the nucleus and the kinetoplast preserved their typical organization. Bars = 1 μm. K, kinetoplast; ht, heterochromatin; nu, nucleolus; m, mitochondrion; f, flagellum. [file 1477-5751-13-11-S3.pdf]
